# C2CD5 in noradrenergic neurons regulates thermogenesis and lipid homeostasis via norepinephrine secretion

**DOI:** 10.1016/j.isci.2026.115446

**Published:** 2026-03-21

**Authors:** Chaitanya K. Gavini, Gwenaël Labouèbe, François Gorostidi, Virginie Mansuy-Aubert

**Affiliations:** 1 Department of Biomedical Sciences, Faculty of Biology and Medicine, University of Lausanne, Lausanne, 1005 Vaud, Switzerland; 2 Department of Otorhinolaryngology-Head and Neck Surgery, Lausanne University Hospital, University of Lausanne, Lausanne, 1005 Vaud, Switzerland

**Keywords:** Physiology, Molecular biology, Neuroscience

## Abstract

Thermogenesis and lipid utilization are important components of energy expenditure; they are regulated within a complex network of behavioral, hormonal, and molecular pathways that collectively maintain energy balance. Norepinephrine (NE) released by sympathetic neurons plays a critical role in modulating these processes. Here, we identify the trafficking protein C2CD5 as a key regulator of NE secretion and thermogenic function. C2CD5 is expressed in dopamine β-hydroxylase (DBH)-positive sympathetic neurons, and its expression is suppressed by obesogenic diets. Using conditional knockout mice lacking C2CD5 in DBH+ neurons, we show that loss of C2CD5 reduces NE secretion, impairs thermogenesis, lowers energy expenditure, and promotes adiposity. These effects are mitigated by NE supplementation. Our findings reveal a functional role for C2CD5 in linking sympathetic tone to systemic metabolic regulation and suggest it may represent a targetable node for metabolic disorders.

## Highlights

• C2CD5 is enriched in DBH+ sympathetic neurons and downregulated by obesogenic diet
• Loss of C2CD5 reduces norepinephrine secretion and impairs BAT thermogenesis
• Conditional knockout mice show decreased energy expenditure and increased adiposity
• Norepinephrine supplementation rescues thermogenic and metabolic defects

## Graphical abstract

**Figure:**
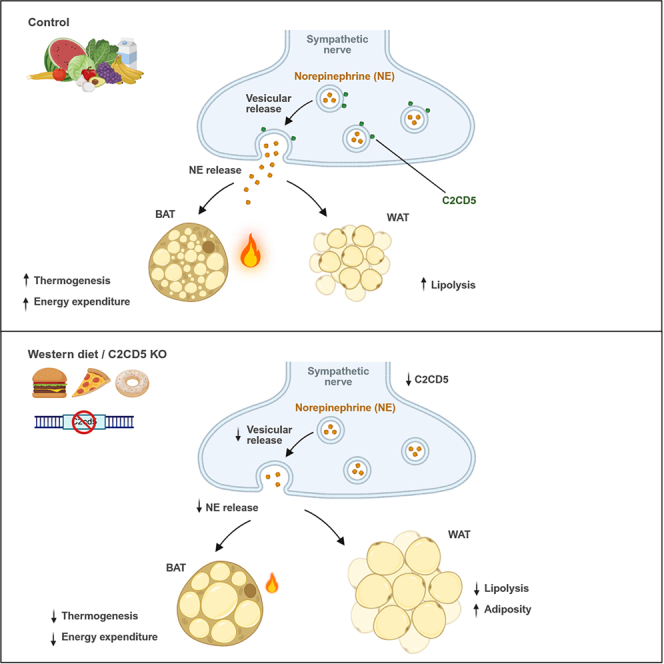

## Introduction

Over the past few decades, the prevalence of obesity has nearly tripled throughout the globe, leading to a significant rise in associated health risks, including type 2 diabetes, hypertension, and reduced life expectancy.^1^^,^^2^ Obesity emerges from a complex interaction between energy intake, physical activity, and energy expenditure, shaped by genetic, social, and environmental cues.^3^^,^^4^ The central nervous system (CNS) is a key regulator of energy homeostasis, integrating these signals to maintain metabolic balance. Even though a considerable amount of research is devoted to understanding the underlying biology of obesity and energy balance, to date it has been of little help to restrain the obesity epidemic. Consequently, innovative therapeutic strategies targeting energy expenditure have gained attention (e.g., GLP1 analogue, semaglutide, which reduces body weight by directly engaging diverse GLP-1 receptor populations and by modulating neural pathways that govern food intake, reward, and energy expenditure).^5^ One such avenue involves stimulating thermogenic activity in brown and white adipose tissues (BAT and WAT), which enhances energy expenditure and holds promise for combating obesity-associated disorders. Notably, sympathetic neuron-mediated norepinephrine (NE) signaling is central to the regulation of adipose tissue thermogenesis, and dysregulation of NE secretion impairs these processes and contributes to metabolic disorders.^6^^,^^7^ However, the proteins governing NE secretion at nerve terminals and their implications in obesity and insulin resistance remain uncharted.

Activation of BAT represents a critical physiological response to cold exposure and excess caloric intake.^8^^,^^9^ BAT dissipates energy in the form of heat through mitochondrial uncoupling, and this process is tightly regulated by sympathetic nervous system (SNS) input.^9^ NE released from sympathetic nerve terminals binds to β-adrenergic receptors on adipocytes, triggering thermogenic pathways that involve the upregulation of UCP1 and increased mitochondrial activity.^10^^,^^11^^,^^12^^,^^13^ Thus, the proper function of sympathetic neurons is essential for thermogenic homeostasis and resistance to obesity.

C2CD5 is a trafficking protein whose expression is reduced in obesity and has been previously implicated in energy homeostasis, particularly through its role in regulating melanocortin-4 receptor (MC4R) trafficking within the hypothalamus.^14^ In addition, C2CD5 has been associated with vesicle trafficking and mitochondrial positioning in neurons.^15^ Despite these findings, the function of C2CD5 within the peripheral SNS, particularly its potential involvement in NE secretion and thermogenic regulation, remains unexplored.

In this study, we uncover a critical role for C2CD5 in sympathetic neurons that produce NE. We demonstrate that C2CD5 is essential for NE secretion, BAT thermogenic function, and energy expenditure. Using a neuron-specific knockout approach, we provide evidence linking C2CD5 to mitochondrial trafficking, NE release, and whole-body metabolic regulation. Collectively, these findings advance our understanding of the neurobiological mechanisms underlying obesity and suggest potential therapeutic avenues targeting sympathetic neuron function to enhance metabolic health.

## Results

### C2CD5 is abundantly expressed in DBH-positive sympathetic neurons and decreases in obesity

**Figure 1:**
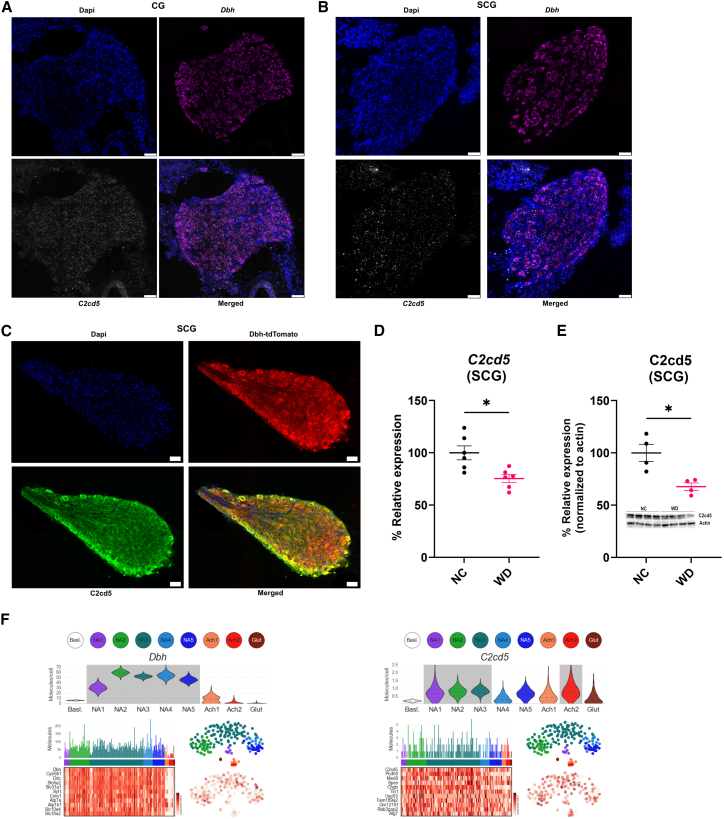
C2CD5 expression in the noradrenergic neurons (A–C) Expression of *C2cd5* and *Dbh* mRNA in celiac (A) and superior cervical ganglia (B), and the expression of C2cd5 in Dbh-tdTomato positive cells of the superior cervical ganglia (C). *n* = 3 biological replicates with 5 sections assessed per replicate. (D–E) mRNA (D) and protein expression (E) of C2cd5 in normal chow (NC) and western diet (WD)-fed mice. *n* = 6/group for mRNA, 4/group (2–3 mice pooled per replicate) for protein. (F) Data generated using single-cell transcriptomic data of mouse thoracic sympathetic ganglia show the average number of detected mRNA molecules expressed by each of the neuronal types in sympathetic ganglia. All data are presented as mean ± S.E.M. Single-group comparisons were conducted using two-tailed t-tests, and *p* < 0.05 was considered statistically significant. ∗*p* < 0.05. NC = normal chow; WD = Western diet. Scale bars, 75 μm (A) and 50 μm (B-C).

C2CD5 is expressed in adipose tissue, where it has been implicated in GLUT4 trafficking, insulin sensitivity, and the browning of adipose tissue.^16^^,^^17^ We previously demonstrated that C2CD5 is broadly expressed throughout the brain, with particularly high enrichment in the hypothalamus,^14^ and that both C2CD5 mRNA and protein levels are reduced in the brain of mice models of diet-induced obesity.^14^ Using whole body knockout of C2CD5 we have shown that loss of C2CD5 leads to the generation of obese phenotype.^14^ In addition, C2CD5 is expressed in NE-producing neurons and is downregulated by an obesogenic diet. Immunofluorescence staining and gene expression analysis revealed that C2CD5 is abundantly expressed in dopamine β-hydroxylase (DBH)-positive sympathetic neurons (Figures 1A–1C). Notably, both C2CD5 mRNA and protein levels were significantly reduced in mice maintained on a Western diet (WD) compared to those fed normal chow (NC), indicating that diet-induced obesity negatively regulates C2CD5 expression (Figures 1D and 1E). To assess the translational relevance of these findings, we analyzed previously published single-cell transcriptomic data from mouse thoracic sympathetic ganglia,^18^ which confirmed C2CD5 expression in DBH-expressing neurons (Figure 1F). Furthermore, immunohistochemical analysis of human beige-like subcutaneous adipose tissue and mouse BAT confirmed C2CD5 expression within adipose depots, supporting a conserved role for C2CD5 in energy balance across human and mouse species (Figure S1).

### Loss of C2CD5 in noradrenergic neurons increases adiposity and alters energy balance

**Figure 2:**
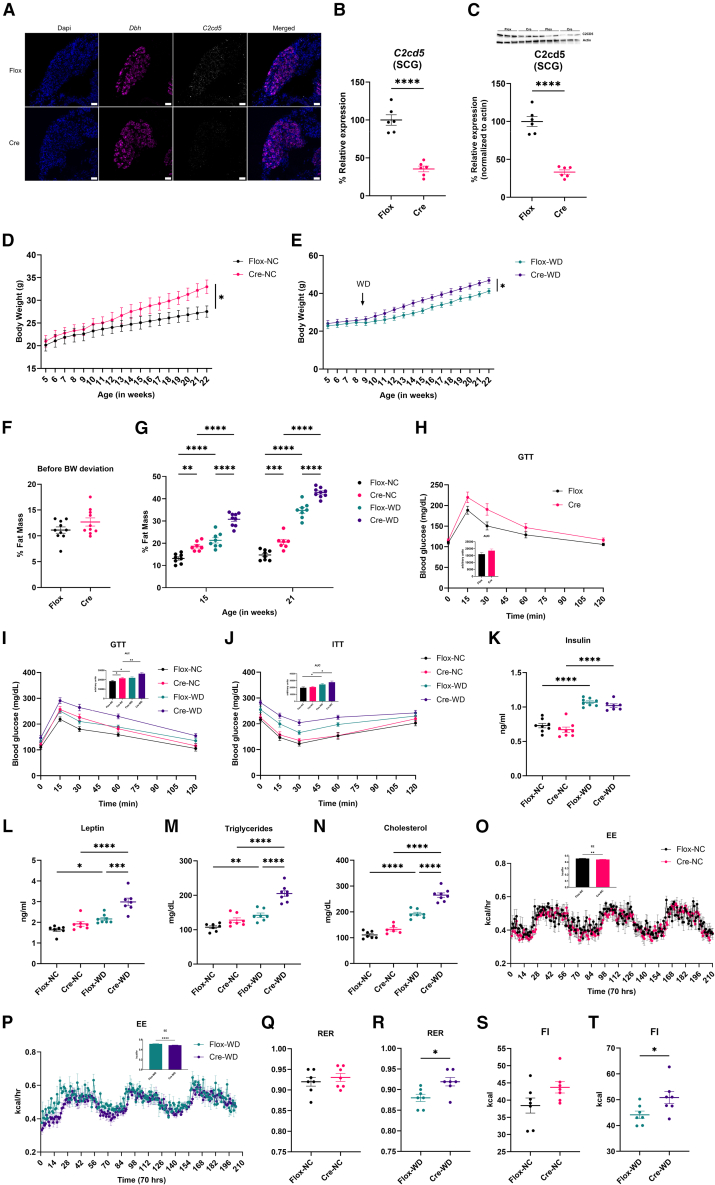
Loss of C2CD5 in DBH+ neurons increases body weight and adiposity (A) ISH shows the loss of *C2cd5* in sympathetic ganglia in Cre mice. *n* = 3 biological replicates with 5 sections assessed per replicate. (B–C) mRNA (B) and protein expression (2–3 mice pooled per replicate) (C) of C2cd5 in Flox and Cre mice. *n* = 6/group. (D–E) Body weight (BW) of Flox and Cre mice under normal chow (NC) (D) and western diet (WD) (E). n = 8–10/group. (F–G) Fat mass percentage before BW deviation (8 weeks of age) (F) and after on WD for 12 weeks (21 weeks of age) (G). n = 7–10/group. (H–I) Glucose tolerance test before BW deviation (8 weeks of age) (H) and after on WD for 10 weeks (I). n = 8–10/group. (J) Insulin tolerance test after on WD for 11 weeks, n = 8–10/group. (K–N) Systemic levels of insulin (K), leptin (L), triglycerides (M), and cholesterol (N). n = 6–8/group. (O–P) Energy expenditure of Flox and Cre mice on NC (O) and WD (P) before BW deviation (8 weeks of age). *n* = 7/group. (Q–R) RER of Flox and Cre mice on NC (Q) and WD (R) before BW deviation (8 weeks of age). *n* = 7/group. (S–T) Food intake of Flox and Cre mice on NC (S) and WD (T) before BW deviation (8 weeks of age). *n* = 7/group. All data are presented as mean ± S.E.M. Single-group comparisons were conducted using a two-tailed t-test, with multiple comparisons analyzed by ANOVA. Repeated measures were analyzed using two-way ANOVA, and *p* < 0.05 was considered statistically significant. ∗*p* < 0.05, ∗∗*p* < 0.005, ∗∗∗*p* < 0.0005, and ∗∗∗∗*p* < 0.00005. NC = normal chow; WD = Western diet. Flox = C2CD5 homozygous floxed, and Cre = C2CD5 homozygous floxed/hemizygous DBH-Cre. Scale bars, 50 μm.

To define the role of C2CD5 in sympathetic neurons, we generated a neuron-specific knockout by crossing C2cd5 flox/flox mice with DBH-Cre transgenic mice, thereby targeting NE-producing neurons. Quantitative PCR and immunostaining of sympathetic ganglia from these mice confirmed an approximate 80% reduction in C2CD5 expression, consistent with recombination efficiency in neurons (Figures 2A–2C).

Conditional knockout mice exhibited significantly increased body weight under both NC and WD conditions compared to littermate controls (Figures 2D and 2E). Body composition analysis showed that the weight gain was primarily attributable to increased fat mass rather than lean mass (Figures 2F and 2G). Glucose tolerance was unaffected before body weight deviation, but had a significant difference after deviation (Figures 2H and 2I). This was not observed during WD (Figures 2H and 2I). Similar results were observed during the insulin sensitivity test (Figures 2J and 2K). However, serum biochemical analyses revealed that WD-fed knockout mice displayed marked metabolic dysregulation, including elevated circulating insulin, leptin, triglycerides, and total cholesterol (Figures 2L–2N). Consistent with obesity-associated lipid overflow due to impaired adipose lipid handling,^19^^,^^20^ histological analysis revealed adipocyte hypertrophy in BAT and WAT (both subcutaneous - inguinal and visceral – epididymal) depots (Figures S2 and S3).

To determine the cause of increased fat accumulation, we performed indirect calorimetry using a Promethion system before the body weight diverged (with dietary shift to WD in the metabolic cages after obtaining baseline observations on NC). Knockout mice had a significant decrease in total energy expenditure in both dietary contexts (Figures 2O–2P). Additionally, under the WD, knockout mice had an elevated respiratory exchange ratio (RER), indicating a shift toward carbohydrate reliance and reduced fat oxidation (Figures 2Q–2R). These mice also showed increased caloric intake, further compounding the energy imbalance (Figures 2S–2T). These data indicate that mice lacking C2CD5 in the DBH-positive cells exhibit higher and specific adiposity with larger adipocytes. Given the established role of SNS β-adrenergic signaling in increasing BAT thermogenesis, glucose uptake, and stimulating lipolysis in all fat depots.^21^ Decreased SNS inputs on BAT or WAT led to an increase in adiposity and less energy or heat produced.^21^ These data suggest that the lack of C2CD5 in the DBH-expressing cells enhances the negative effect of WD and favors weight gain, generating obese phenotypes likely due to a blunted sympathetic input on adipose tissues and decreased thermogenesis.

### C2CD5 regulates NE release in sympathetic ganglia

**Figure 3:**
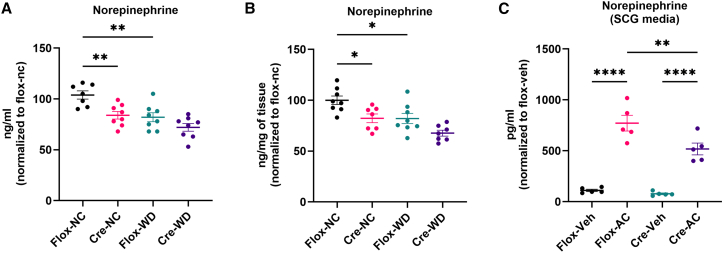
Loss of C2CD5 in DBH neurons decreases NE secretion (A–B) Norepinephrine levels in systemic circulation (A) and in brown adipose tissue (B). n = 7–8/group. (C) Norepinephrine secretion by sympathetic ganglia from Flox and Cre mice. *n* = 5/group. All data are presented as mean ± S.E.M. Multiple comparisons were analyzed by ANOVA. Repeated measures were analyzed using two-way ANOVA, and *p* < 0.05 was considered statistically significant. ∗*p* < 0.05, ∗∗*p* < 0.005, and ∗∗∗∗*p* < 0.00005. NC = normal chow; WD = Western diet. Flox = C2CD5 homozygous floxed, and Cre = C2CD5 homozygous floxed/hemizygous DBH-Cre. Veh = vehicle, AC = acetylcholine chloride.

Adipose tissue is innervated by the SNS, whose activation is essential for lipolysis.^22^ NE, the principal SNS neurotransmitter, is synthesized and stored in synaptic vesicles of sympathetic fibers^13^ and, upon SNS activation, is released into the synaptic cleft to act on α- and β-adrenergic receptors, thereby promoting lipolysis.^23^^,^^24^ We observed an increase in fat mass and decreased energy expenditure in mice lacking C2CD5 from DBH neurons, suggesting that NE secretion may be impaired. Given the role of NE in promoting lipolysis and thermogenesis, we measured NE concentrations in plasma and BAT. Knockout mice exhibited significantly lower NE levels in both compartments compared to controls (Figures 3A and 3B). To investigate whether C2CD5 is required for stimulus-coupled NE release, we cultured sympathetic ganglia *ex vivo* and stimulated them with acetylcholine (AC). AC is released from pre-ganglionic fibers to the efferent SCG and stimulates NE release to target tissues.^23^ NE secretion was markedly impaired in ganglia from knockout mice, indicating that C2CD5 is required for effective NE exocytosis (Figure 3C). These results show that loss of C2CD5 in DBH-expressing cells of the sympathetic ganglia diminishes NE release and probably plays a role in decreasing the activity of metabolically active tissue such as BAT.

### Impaired thermogenic adaptation in C2CD5-deficient sympathetic neurons

**Figure 4:**
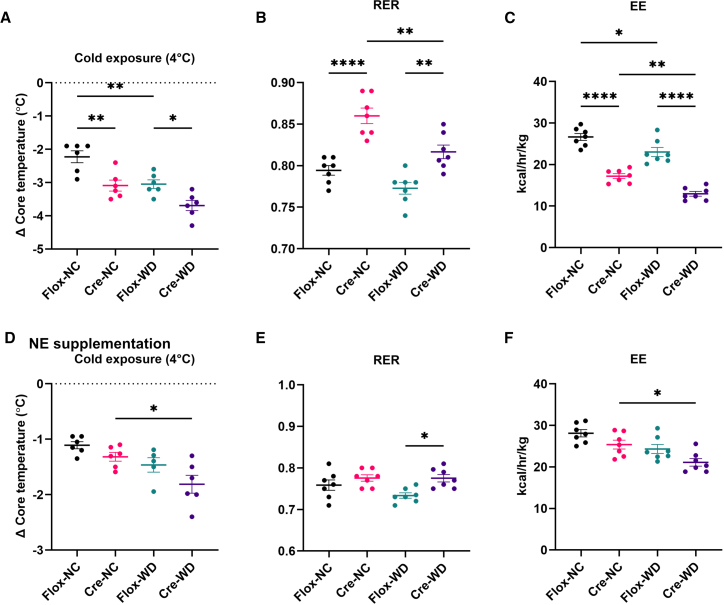
Loss of C2CD5 in DBH neurons decreases response to cold (A–C) Changes in core body temperature (A), RER (B), and energy expenditure (C) when exposed to 4°C in Flox and Cre mice on NC and WD. n = 6–7/group. (D–F) Changes in core body temperature (D), RER (E), and energy expenditure (F) when exposed to 4°C of Flox and Cre mice on NC and WD after norepinephrine supplementation. n = 6–7/group. All data are presented as mean ± S.E.M. Multiple comparisons were analyzed by ANOVA, and *p* < 0.05 was considered statistically significant. ∗*p* < 0.05, ∗∗*p* < 0.005, and ∗∗∗∗*p* < 0.00005. NC = normal chow; WD = Western diet. Flox = C2CD5 homozygous floxed, and Cre = C2CD5 homozygous floxed/hemizygous DBH-Cre.

SNS is critical for regulating energy expenditure by controlling resting metabolic rate and initiating thermogenesis in response to physiological stimuli such as cold exposure.^21^ In BAT, β3-adrenergic receptor activation during cold exposure drives thermogenesis via mitochondrial uncoupling.^24^ Studies using both human and rodent models show behavioral and physiological responses to cold exposure by decreasing heat dissipation and increasing heat generation via BAT thermogenesis.^25^ This upregulation of BAT thermogenesis is achieved by increasing the activity of the sympathetic nerve that innervates the BAT, releasing NE to favor β-oxidation and UCP1 activity.^10^ To evaluate thermogenic function, we subjected mice to cold exposure. Knockout animals exhibited a significantly lower core body temperature than controls (Figure 4A). Indirect calorimetry during cold exposure revealed decreased energy expenditure and elevated RER in the knockout group, indicative of impaired BAT activity and reduced fat oxidation (Figures 4B and 4C). Notably, systemic NE administration rescued these thermogenic defects, normalizing core temperature, RER, and energy expenditure in knockout mice (Figures 4D–4F), further underscoring the critical role of C2CD5 in NE-dependent thermogenesis. Collectively, these data demonstrate that C2CD5 in DBH-expressing sympathetic neurons is required for proper sympathetic drive to adipose tissue and maintenance of body weight.

## Discussion

This study identifies C2CD5 as a key regulator of thermogenesis and lipid metabolism through its role in NE secretion from sympathetic neurons. By impairing NE release, C2cd5 deletion compromises BAT activation and energy expenditure, promoting adiposity even under non-obesogenic conditions. Our findings place C2CD5 at a critical intersection of vesicular trafficking and sympathetic neuron function. The observed reductions in NE release likely stem from disrupted trafficking of vesicles or organelles, including mitochondria, which are essential for neuronal activity and neurotransmitter secretion.^26^^,^^27^ This aligns with prior evidence implicating C2CD5 in mitochondrial dynamics,^15^ adding functional relevance to its presence in sympathetic neurons. Zhou et al.^16^ reported that whole-body C2CD5 deficiency reduces thermogenesis, decreases energy expenditure, and increases susceptibility to obesity, effects attributed to impaired catecholamine secretion from the adrenal gland. Because DBH is expressed in both sympathetic neurons and adrenal chromaffin cells, the DBH-Cre-mediated deletion employed here likely disrupts catecholamine secretion in the same compartments. The striking similarity between the metabolic and thermogenic phenotypes observed in our conditional knockout mice and those reported in global knockouts confirms the essential role of C2CD5. Importantly, our neuron-specific approach demonstrates that loss of C2CD5 within the catecholaminergic system alone is sufficient to recapitulate these phenotypes, indicating that impaired catecholamine handling, rather than C2CD5 deficiency in peripheral tissues such as adipocytes, is the primary driver of thermogenic and metabolic defects. Collectively, these data refine previous conclusions by localizing a critical role for C2CD5 within DBH-expressing cells in regulating adaptive thermogenesis and energy expenditure, highlighting its central importance in sympathetic neuron-mediated metabolic control.

The downregulation of C2CD5 by WD highlights a maladaptive response that could further promote metabolic disease. Reduced expression of C2CD5 in NE-producing neurons may impair their ability to respond to thermogenic cues, contributing to energy imbalance and weight gain. These changes occurred even in the absence of glucose intolerance, emphasizing that lipid handling and energy expenditure are more directly affected. Moreover, the rescue of thermogenic phenotypes through NE supplementation confirms that NE deficiency is central to the observed defects. This suggests that strategies to restore NE signaling, or enhance C2CD5 function, could mitigate metabolic disturbances. It also opens avenues for pharmacological targeting of neuronal trafficking pathways to stimulate energy expenditure and treat obesity. Future work should aim to delineate the molecular mechanisms by which C2CD5 governs NE secretion. Identifying interacting partners, downstream effectors, and potential modifiers of its activity will be of interest. Although SNS overactivity often accompanies obesity as a compensatory response, chronic SNS stimulation can promote metabolic syndrome by driving obesity, hyperglycemia, insulin resistance, and hypertension.^21^^,^^28^ Recent clinical trials targeting central sympathetic activity show promise for improving metabolic control in at-risk patients.^28^ Determining whether C2CD5 function can be selectively enhanced in sympathetic neurons without adverse effects may inform translational approaches.

While NE and SNS axons are major regulators of thermogenesis and lipid mobilization (especially in brown and beige adipose tissues), they are not the sole neural influences.^29^^,^^30^^,^^31^^,^^32^ Recent research demonstrates that additional neural pathways, including parasympathetic, sensory, and somatosensory innervation, also contribute to adipose tissue regulation and energy expenditure.^33^^,^^34^^,^^35^ For example, sensory nerves impact local adipose tissue physiology, and hormones such as thyroid hormone modulate thermogenic responses independently of sympathetic activation.^35^^,^^36^ This reflects the complexity of metabolic regulation.

The systemic nature of DBH+ cell targeting means that the metabolic phenotype observed could result from combined effects on multiple organ systems innervated by sympathetic neurons, not exclusively from adipose tissue modulation. Such a global DBH+ knockout includes sympathetic neurons innervating various tissues, potentially affecting cardiovascular, gastrointestinal, and other systems regulated by sympathetic tone^37^^,^^38^^,^^39^^,^^40^; these off-target effects represent an important caveat. Interpretation is limited by this, as effects on energy balance and lipid metabolism may be confounded by changes elsewhere in the body’s sympathetic network. While our NE supplementation experiments partially rescue the metabolic phenotype, which supports NE’s central role, they do not fully exclude contributions from other systems influenced by DBH+ neuron C2CD5 deletion, highlighting the need for future studies to dissect peripheral versus central and tissue-specific actions of C2CD5 within the SNS.

C2CD5 is essential for proper NE secretion from sympathetic neurons, enabling thermogenic and metabolic responses critical for maintaining energy balance. Loss of C2CD5 disrupts the NE-mediated activation of BAT, reduces energy expenditure, and promotes lipid accumulation. Our findings raise the possibility that modulating C2CD5 function in the SNS could impact energy expenditure and adiposity. Further investigation will be needed to assess the translational feasibility of targeting C2CD5 for therapeutic benefit.

### Limitations of the study

Our genetic strategy targets DBH-expressing cells broadly, including sympathetic neurons and adrenal chromaffin cells, and therefore does not allow precise dissection of circuit or tissue-specific contributions. However, the rescue of thermogenic defects by NE administration supports a primary role for catecholamine deficiency. The cellular mechanisms by which C2CD5 regulates NE secretion were not directly resolved, although our functional data provide clear evidence for impaired activity-dependent release. Systemic metabolic outcomes may also reflect indirect effects from other sympathetically regulated organs. Finally, while C2CD5 expression is confirmed in human adipose tissue, functional validation in human sympathetic systems remains an important goal for future studies.

## Resource availability

### Lead contact

Further information and requests for resources and reagents should be directed to the Lead contact, Virginie Mansuy-Aubert (virginie.mansuy-aubert@unil.ch).

### Materials availability

This study did not generate new unique reagents.

### Data and code availability

• Data: All data supporting the findings of this study are available within the paper and its supplemental information.
• Code: No code was used in this study, and there is no original code to report.
• Other items: Any additional information required to reanalyze the data reported in this paper is available from the lead contact upon request.

## Acknowledgments

We thank Tiemin Liu, Department of Internal Medicine, Hypothalamic Research Center, University of Texas Southwestern Medical School, Dallas, Texas, for helping with mouse generation. We thank the University of Lausanne animal facility for mouse housing. Funds were provided by 10.13039/501100006390University of Lausanne to VM-A.

## Author contributions

CKG, conception and design, acquisition of data, analysis and interpretation of data, drafting, revising, and approving the article. GL, acquisition of data, revising, and approving the article. FG, Acquisition of data, revising, and approving the article. VM-A, conception and design, analysis and interpretation of data, drafting, revising, and approving the article.

## Declaration of interests

The authors declare no competing interests.

## STAR★Methods

### Key resources table

**Table:** 

REAGENT or RESOURCE	SOURCE	IDENTIFIER
**Antibodies**		

C2CD5 (KIAA0528)	Bethyl laboratories	Cat#A301-469A; RRID:AB_999597
Beta-Actin	Abcam	Cat#ab8226; RRID:AB_306371
Goat Anti-Rabbit IgG H&L Alexa Fluor® 488	Abcam	Cat#ab150077; RRID:AB_2630356

**Biological samples**		

Human adipose tissue (subcutaneous supraclavicular depot)	Center Hospitalier Universitaire Vaudois (Lausanne)	N/A

**Chemicals, peptides, and recombinant proteins**		

Nerve Growth Factor	Sigma	N8133
MEM, HEPES, no glutamine	ThermoFisher	32360026
GlutaMAX	ThermoFisher	35050038
HBSS	ThermoFisher	14025092
D-(+)-Glucose	ThermoFisher	A16828.36
Acetylcholine Chloride	Sigma	A6625
Insulin (Actrapid 100IE/UI/ml)	Novo Nordisk	N/A
Triton X-100	Sigma	X-100
SDS	Fisher bioreagents	BP1311-1
protease/phosphatase inhibitor	ThermoFisher	A32961
Norepinephrine	Sigma	A9512
PFA	Sigma	P6148
BSA	Sigma	A7906
Horse Serum	ThermoFisher	26050088

**Critical commercial assays**		

NE ELISA kit	Abcam	AB287789
RNAscope fluorescent multiplex reagents	Advanced Cell Diagnostics	323270
Insulin	Millipore	EZRMI
Triglycerides	Sigma	MAK266
Cholesterol	Sigma	MAK043
Leptin	Millipore	EZRML
Mouse and Rabbit specific HRP/DAB detection IHC kit	Abcam	AB64264
FastStart Universal SYBR Green Master (Rox)	Roche	4913914001
High-Capacity cDNA Reverse Transcription Kit	ThermoFisher	4374967

**Experimental models: Organisms/strains**		

C57BL/6J-C2CD5tm2Vma/J (C2CD5-flox)	This paper	N/A
Tg(Dbh-icre)1Gsc	Dr. Anne Vassalli (University of Lausanne)	MGI:4355551
B6.129 (Cg)-Gt(ROSA)26Sortm14(CAG-tdTomato)Hze/J	Dr. Fanny Langlet (University of Lausanne)	IMSR_JAX:007908

**Oligonucleotides**		

C2cd5 (for ISH)	Advanced Cell Diagnostics	436771
Dbh (for ISH)	Advanced Cell Diagnostics	407851-C2
C2cd5-F (5′-3′): GCG GAG AAA TCA ATG TTG TGG T	This paper	N/A
C2cd5-R (5′-3′): ATC AAT CCA CTG ATA CTC GGG A	This paper	N/A
Beta-actin-F (5′-3′): AGA GGG AAA TCG TGC GTG AC	This paper	N/A
Beta-actin-R (5′-3′): CAA TAG TGA TGA CCT GGC GT	This paper	N/A

**Software and algorithms**		

FIJI/ImageJ	https://imagej.net/software/fiji/#publication	RRID:SCR_002285
Graphpad prism	GraphPad	RRID:SCR_002798
Thunder imaging systems	Leica	RRID:SCR_023794

**Other**		

Cell Culture Inserts	Millipore	PICM01250
Contour next glucometer	VitaServ	7830311
Contour Next sensors	VitaServ	5225293
4–15% Mini-PROTEAN Precast Protein Gels	Bio-Rad	4561086DC
Promethion metabolic cages	Sable systems	N/A
PVDF membranes	Bio-Rad	1620177
Standard chow (NC)	Safe diets	Safe-150
Western diet (WD)	Envigo	TD88137
Portable Digital Thermometer including Probe	Harvard Apparatus	34–1401
Body Composition Mice Analyzer	Bruker	MiniSpec LF50

### Experimental model and study participant details

#### Animal care and use

C2CD5 floxed mice were generated at University of Lausanne and crossed with DBH-Cre mice (kindly provided by Dr. Anne Vassalli, University of Lausanne) and with tdTomato mice (kindly provided by Dr. Fanny Langlet, University of Lausanne). All animal procedures were conducted in accordance with the guidelines of the Service de la consommation et des Affaires vétérinaires (SCAV) of the Canton de Vaud, Switzerland, and the Direction générale de l’agriculture, de la viticulture et des affaires vétérinaires (DGAV) – Affaires vétérinaires, Protection des animaux (cantonal approval #VD3814). Mice with identical genetic backgrounds were compared in all experiments. Genotyping was performed for all animals, and protein knockout was confirmed by immunofluorescence and Western blotting. To evaluate the phenotype of littermates, homozygous floxed C2CD5 are bred with homozygous floxed C2CD5/hemizygous DBH-cre, the littermates compared are homozygous floxed C2CD5 called Flox, and homozygous floxed C2CD5/hemizygous DBH-cre called Cre. The presence of the Cre expression did not modify the phenotype in previous cohorts (not shown). Mice (male) were housed 4–5 per cage under a 12:12 h light/dark cycle and received either normal chow (NC; SAFE 150) or Western diet (WD; TD88137, Teklad Diets; 42% kcal from fat, 34% sucrose by weight, 0.2% cholesterol; Envigo) for 12 weeks following initial assessment of energy balance and glucose homeostasis (∼week 8–9, prior to body weight divergence). Body weight was recorded weekly from weaning, and body composition was measured at regular intervals using NMR (Bruker). All metabolic experiments were done exclusively using male mice littermates to avoid confounding effect of hormones with the experimenter blinded to genotype and treatment, following ARRIVE guidelines.

#### Human subjects

Informed consent was obtained from all participants (3 females; 30, 32, and 58 years old) prior to collection of subcutaneous supraclavicular adipose tissue. All procedures were conducted in accordance with the study protocol, Swiss legal requirements, the Declaration of Helsinki, and established principles of research integrity. The study was approved by the Ethical Committee of the Canton of Vaud (protocol #CER-VD BASEC ID: 2023-01412).

#### Ethics

All animal experiments were conducted in strict accordance with the guidelines and regulations of the Service de la consommation et des affaires vétérinaires (SCAV) of the Canton of Vaud, Switzerland, and the Direction générale de l’agriculture, de la viticulture et des affaires vétérinaires (DGAV) – Affaires vétérinaires, Protection des animaux. Every effort was made to minimize animal suffering. Collection of human samples was performed in full compliance with the approved study protocol, Swiss legal requirements, the current version of the World Medical Association Declaration of Helsinki, and established principles governing integrity in scientific research involving human participants.

### Method details

#### Superior cervical ganglia (SCG)/Celiac ganglia (CG) organotypic culture

Juvenile male flox and Cre mice (4-6weeks) were anesthetized with isoflurane and decapitated, and superior cervical ganglia (SCG) and celiac ganglia (CG) were rapidly dissected and cultured on air-interface membranes (Millipore). Explants were maintained for 24 h in standard culture medium supplemented with nerve growth factor (Sigma)^41^ at 37°C in 5% CO_2_ prior to treatment. Cultures were then exposed to 10 mM acetylcholine chloride (Sigma) or vehicle for 2 h, after which norepinephrine (NE) levels in both explants and media were quantified using a mouse NE ELISA kit (MyBioSource, Mouse NE Elisa kit) according to the manufacturer’s instructions.

#### Glucose and insulin tolerance tests

Glucose and insulin tolerance testing were performed as previously described.^14^^,^^42^ For glucose tolerance, mice fasted overnight (12 h) received an intraperitoneal (i.p.) injection of glucose (1 g/kg body weight; Thermo Scientific) after baseline glucose measurement, and blood glucose was monitored at 15, 30, 60, and 120 min using a Contour Next glucometer (VitaServ). For insulin tolerance, mice fasted for 4 h were injected i.p. with insulin (0.5 U/kg body weight; Actrapid, Novo Nordisk) and blood glucose levels were measured at the same time points.

#### Western blotting

Protein isolation and western blotting were performed as described before.^14^^,^^15^ Briefly, tissues were homogenized in ice cold lysis buffer (1XPBS +1% Triton X-100 + 0.01% SDS + protease/phosphatase inhibitor). Equal amounts of protein (20 μg) were resolved on 4–15% Mini-PROTEAN precast gels (Bio-Rad) and transferred onto PVDF membranes (Bio-Rad). Membranes were probed with antibodies against actin (Abcam, #ab8226) and C2CD5 (Bethyl Laboratories, #A301-469A) at the manufacturers’ recommended dilutions. Two to three mice were pooled per biological replicate. Immunoblots were quantified using FIJI, normalized to actin, and expressed relative to control groups (set as 100%).

#### Metabolic cage analysis

Metabolic measurements were conducted using Promethion metabolic cages (Sable Systems) in Flox and Cre mice prior to body weight divergence to avoid confounding effects of weight on energy balance.^14^^,^^43^ Energy expenditure and related parameters were also assessed during cold exposure, with or without norepinephrine (NE) challenge. Mice were individually housed in clean cages with access to water but no food to eliminate cold-induced hyperphagia and diet-induced thermogenesis. Core body temperature was measured rectally before and after cold exposure using a probe thermometer (Kent Scientific). For NE challenge, mice received an intraperitoneal injection of NE (Sigma) 30 min prior to cold exposure at a dose of 2.53 ∗ body mass ˆ (−0.4),^21^ and metabolic responses were monitored in individual chambers.

#### *In situ* hybridization

Fluorescence *in situ* hybridization (FISH) was performed on 20 μm fresh-frozen tissue sections using RNAscope fluorescent multiplex reagents (Advanced Cell Diagnostics) following the manufacturer’s instructions. Tissue sections were hybridized with RNA probes for C2cd5 and Dbh, and signal amplification was achieved using the multiplex reagents. Nuclei were counterstained with DAPI, and sections were mounted with antifade medium. Images were acquired on a THUNDER Imaging System (Leica Microsystems) and analyzed using ImageJ software.

#### Serum triglycerides, cholesterol, insulin, leptin, and NE measurement

Serum from NC and WD mice were processed for triglycerides (Sigma), cholesterol (Sigma), insulin (EMD Millipore), leptin (R&D systems), and NE (MyBioSource) using manufacturer’s instructions.

#### Histology

Histochemical analysis was performed on formalin-fixed paraffin embedded mouse brown, subcutaneous, and visceral adipose tissue. H&E staining was performed on 4 μm thick sections as previously reported.^14^^,^^42^

#### Immunofluorescence/immunohistochemistry

Tissues were dissected and either flash-frozen or processed for paraffin embedding, as previously described.^14^^,^^42^ For frozen sections, 20 μm slices were mounted on glass slides, fixed in 4% PFA, and washed with 1× PBS. Sections were permeabilized and blocked in 5% BSA with 0.2% Triton X-100 in PBS for 1 h at room temperature. Primary antibody against C2CD5 (Bethyl Laboratories) was applied at 1:400 overnight at 4°C, followed by 2 h incubation with secondary antibody. Nuclei were counterstained with DAPI, and sections were mounted with antifade medium. Imaging was performed on a THUNDER Imaging System (Leica Microsystems), and analysis was conducted using ImageJ.

For paraffin-embedded sections, slides were deparaffinized and rehydrated through sequential washes in xylene, 100%, 95%, and 70% ethanol, and deionized water. Antigen retrieval was performed using heat-mediated treatment in 10 mM sodium citrate buffer (pH 6.0), followed by immunodetection using a mouse- and rabbit-specific HRP/DAB IHC kit (Abcam) according to the manufacturer’s instructions.

#### Quantitative PCR

Total RNA was extracted from tissue using the Arcturus PicoPure RNA Isolation Kit (ThermoFisher) and reverse-transcribed into cDNA with the High-Capacity cDNA Reverse Transcription Kit (ThermoFisher). Quantitative PCR (qPCR) was performed using an SYBR Green–based assay (Roche) with primers from Microsynth. Gene expression was normalized to 18S rRNA, and relative quantification was calculated using the ΔΔCT method, with the control group set to 100%.

### Quantification and statistical analysis

Data are presented as mean ± SEM. Statistical analyses were performed using GraphPad Prism 10.4.1. Single-group comparisons were conducted using one- or two-tailed t-tests as appropriate, with multiple comparisons analyzed by ANOVA. Repeated measures were analyzed using two-way ANOVA, and *p* < 0.05 was considered statistically significant. ∗*p* < 0.05, ∗∗*p* < 0.005, ∗∗∗*p* < 0.0005, ∗∗∗∗*p* < 0.00005.
