# Supplementary material for: C2CD5 in noradrenergic neurons regulates thermogenesis and lipid homeostasis via norepinephrine secretion
Source: iScience. 2026 Mar 21;29(4):115446. doi: 10.1016/j.isci.2026.115446 (PMC13090686; doi:10.1016/j.isci.2026.115446)
Supplement: Document S1. Figures S1–S3 [file mmc1.pdf]

iScience, Volume *29*

Supplemental information

C2CD5 in noradrenergic neurons regulates thermogenesis and lipid homeostasis via norepinephrine secretion

Chaitanya K. Gavini, Gwenaël Labouèbe, François Gorostidi, and Virginie Mansuy-Aubert

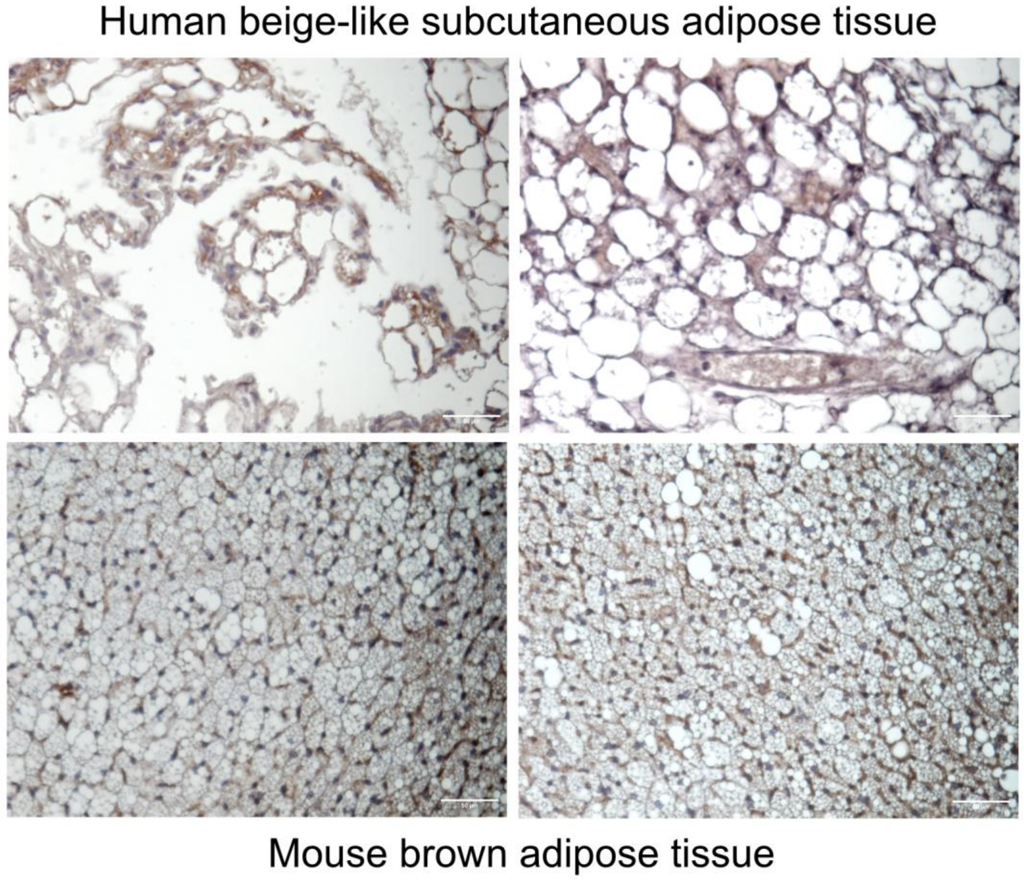

Supplementary Figure 1: Expression of C2CD5 in human (subcutaneous supraclavicular depot) and mouse brown adipose tissue**.** Scale 50µm.

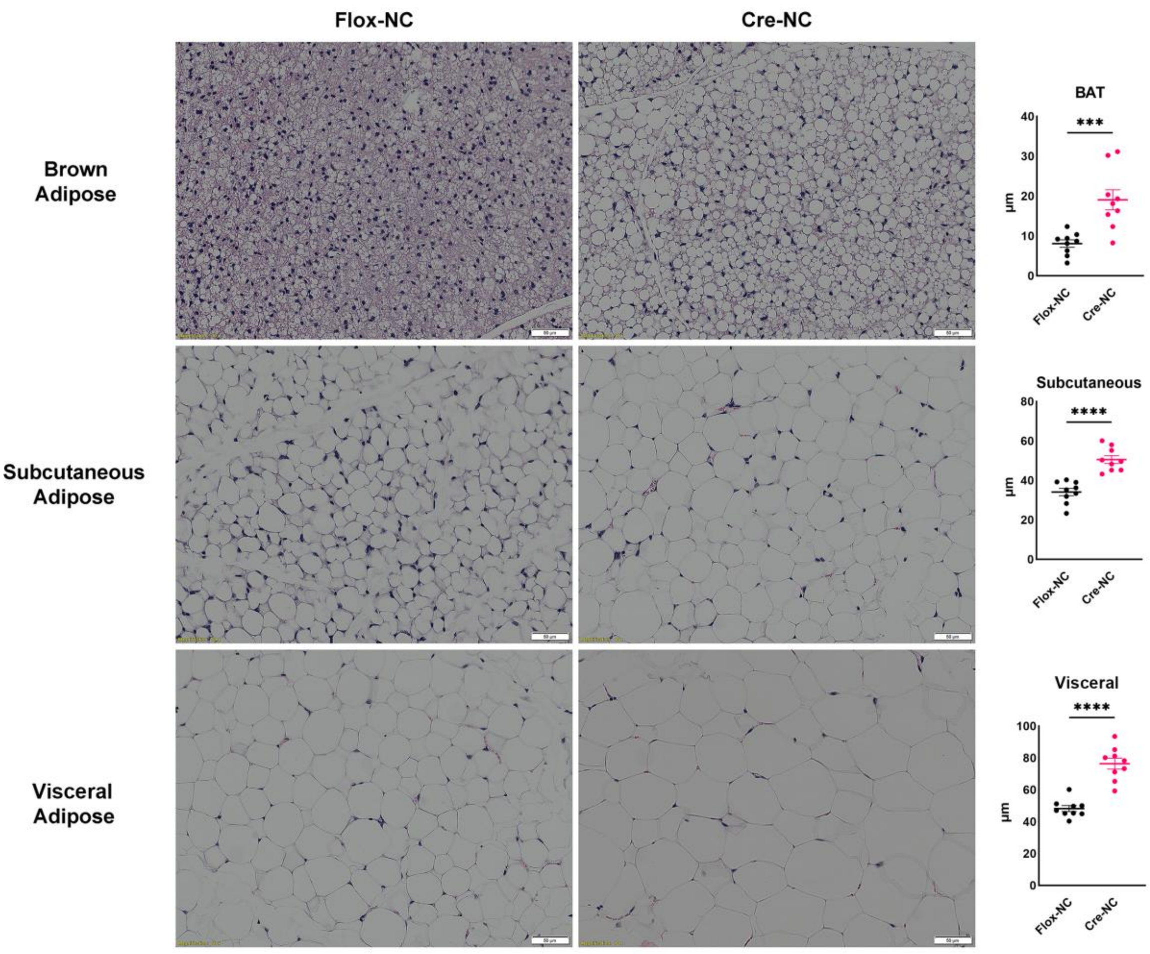

Supplementary Figure 2: Loss of C2CD5 in DBH neurons increases lipid accumulation in adipose tissue on NC. H&E staining of brown, subcutaneous (inguinal), and visceral (epididymal) adipose tissue from flox and cre mice fed NC showing increased adiposity (as measured by cell diameter in µm) in the cre mice compared to flox mice. Scale 50µm. Quantification is average adipocyte size per section (n= 3 sections/mice x 3 mice/group = 9 sections/group). All data are mean±S.E.M. ***$p<0.0005$, ****$p<0.00005$.

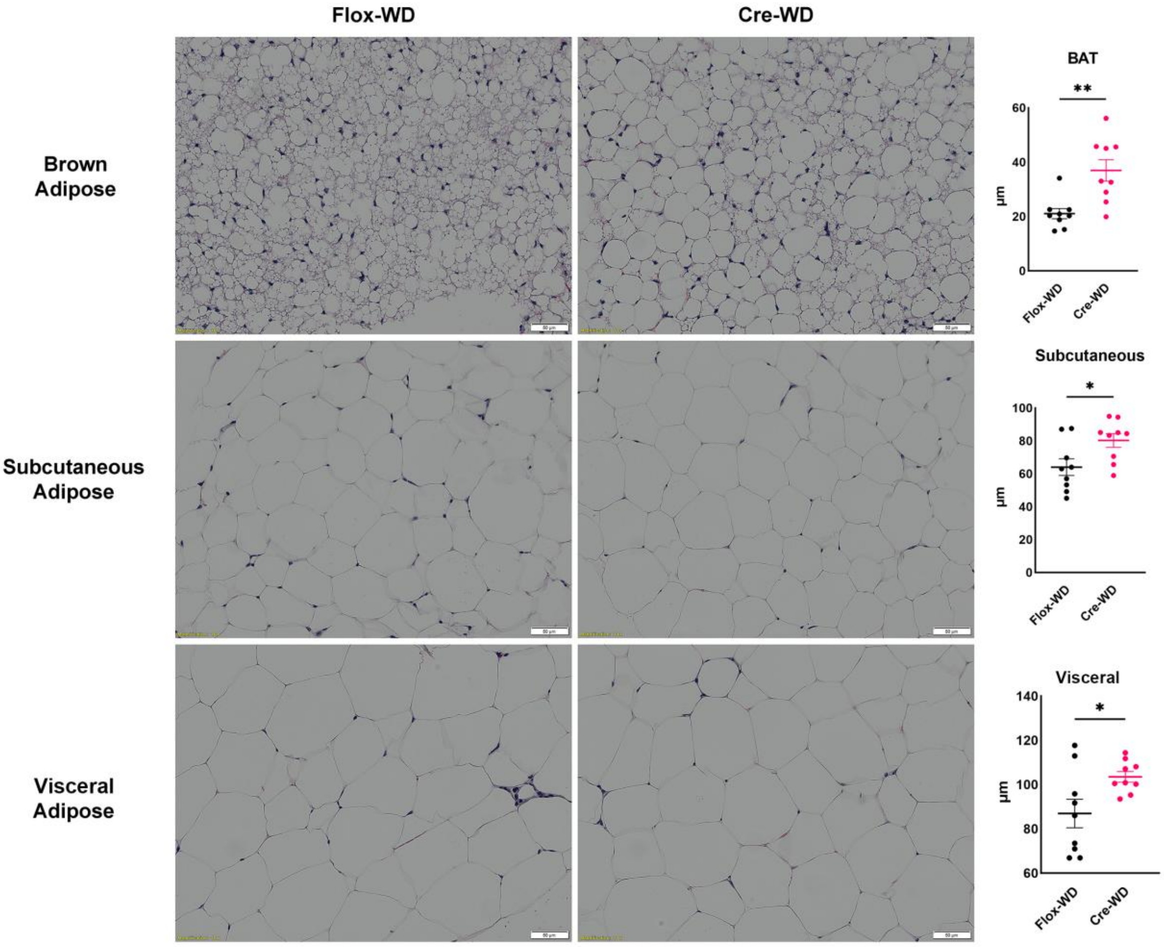

Supplementary Figure 3: Loss of C2CD5 in DBH neurons increases lipid accumulation in adipose tissue on WD feeding. H&E staining of brown, subcutaneous (inguinal), and visceral (epididymal) adipose tissue from flox and cre mice fed WD showing increased adiposity (as measured by cell diameter in μm) in the cre mice compared to flox mice. Scale 50μm. Quantification is average adipocyte size per section (n= 3 sections/mice x 3 mice/group = 9 sections/group). All data are mean±S.E.M. *p<0.05, **p<0.005.
